# Organic Air Quality Markers of Indoor and Outdoor PM_2.5_ Aerosols in Primary Schools from Barcelona

**DOI:** 10.3390/ijerph17103685

**Published:** 2020-05-23

**Authors:** Barend L. van Drooge, Ioar Rivas, Xavier Querol, Jordi Sunyer, Joan O. Grimalt

**Affiliations:** 1 Institute of Environmental Assessment and Water Research (IDAEA-CSIC), Jordi Girona 18-26, 08034 Barcelona, Spain; ioar.rivas@isglobal.org (I.R.); xavier.querol@idaea.csic.es (X.Q.); joan.grimalt@idaea.csic.es (J.O.G.); 2 Barcelona Institute for Global Health (ISGlobal), Dr. Aiguader 88, 08003 Barcelona, Spain; jordi.sunyer@isglobal.org

**Keywords:** primary schools, polycyclic aromatic hydrocarbons, TRAP, indoor air quality, playground, organic aerosol

## Abstract

Airborne particulate matter with an aerodynamic diameter smaller than 2.5 µg, PM_2.5_ was regularly sampled in classrooms (indoor) and playgrounds (outdoor) of primary schools from Barcelona. Three of these schools were located downtown and three in the periphery, representing areas with high and low traffic intensities. These aerosols were analyzed for organic molecular tracers and polycyclic aromatic hydrocarbons (PAHs) to identify the main sources of these airborne particles and evaluate the air quality in the urban location of the schools. Traffic emissions were the main contributors of PAHs to the atmospheres in all schools, with higher average concentrations in those located downtown (1800–2700 pg/m^3^) than in the periphery (760–1000 pg/m^3^). The similarity of the indoor and outdoor concentrations of the PAH is consistent with a transfer of outdoor traffic emissions to the indoor classrooms. This observation was supported by the hopane and elemental carbon concentrations in PM_2.5_, markers of motorized vehicles, that were correlated with PAHs. The concentrations of food-related markers, such as glucoses, sucrose, malic, azelaic and fatty acids, were correlated and were higher in the indoor atmospheres. These compounds were also correlated with plastic additives, such as phthalic acid and diisobutyl, dibutyl and dicyclohexyl phthalates. Clothing constituents, e.g., adipic acid, and fragrances, galaxolide and methyl dihydrojasmonate were also correlated with these indoor air compounds. All these organic tracers were correlated with the organic carbon of PM_2.5_, which was present in higher concentrations in the indoor than in the outdoor atmospheres.

## 1. Introduction

Urban atmospheric particulate matter (PM) is a highly complex mixture whose composition depends on size, sources, weather, transport, insolation and other conditions. Airborne particles with an aerodynamic diameter lower than 2.5 µm (PM_2.5_) are mainly constituted of carbonaceous materials, including elemental carbon (EC) and organic carbon (OC) [1,2] and contain thousands of organic compounds that contribute to their toxic potential [3,4,5]. Combustion by-products generated during fossil fuel or biomass burning are responsible for a significant fraction of these toxic compounds, such as polycyclic aromatic hydrocarbons (PAHs) [6,7,8] which may be found both in outdoor and indoor atmospheres [9,10].

Epidemiological and toxicological studies have described several deleterious health effects associated with PM exposure, namely inflammatory, respiratory and cardiovascular impairments, neurodevelopment disorders, cataracts, diabetes and chronic diseases [11,12,13,14,15]. The World Health Organization estimates that ambient air pollution is responsible for 3.7 million premature deaths per year worldwide [16].

Cities are the environments of highest exposure of large populations to the effects of these toxic compounds. Many activities developed in their streets and buildings involve atmospheric PM release which is subsequently inhaled by the inhabitants, including children. Barcelona is a densely populated city that has a high density of motorized traffic and a large proportion of diesel vehicles, which results in a dominant influence of road traffic-related air pollutants (TRAP) on the local air quality [6,17,18].

Children are sensitive to the effects of environmental pollutants because they are in a development stage [19,20]. Chemical insults in utero and in early infancy can result in disease and disability across the lifespan [21]. A large proportion of children’s lifetime is spent at school for education and social activities. Remarkably, the impact of urban pollution in school environments has received limited attention, particularly concerning airborne organic pollutants [22,23]. Outdoor traffic emissions, measured as EC, were found to be dominant sources in the indoor atmospheres of the schools, while OC was also an important contributor to the indoor PM_2.5_. Nevertheless, the origin of the organic material was not resolved, and toxic organic compounds, such as PAH, were not analyzed [23]. Therefore, further insight on the chemical composition of the inhalable particles in schools is needed both in outdoor and indoor environments.

The EU BREATHE project (BRain dEvelopment and Air polluTion ultraparticles in scHool childrEn) aims at studying the impact of urban air pollution on the cognitive development of schoolchildren. An extensive air measurement campaign in 39 primary schools was carried out in the metropolitan area of Barcelona in 2012 and 2013 which involved PM_2.5_ sampling indoor (classroom) and outdoor (playgrounds). The schools selected for study were located downtown, in the area with high traffic, and in the periphery, with lower traffic intensity.

In this context, additional funding from the La Caixa Foundation allowed us to extend the study to the organic constituents of the collected aerosols. Thus, the present paper reports the results of the in-depth analysis of organic molecular tracers and PAHs in the PM_2.5_ aerosols collected in representative schools of areas with different TRAP levels. The results are used to identify the main sources of these airborne particles and to get insight into the processes responsible for the presence of these aerosols in the urban schools located close to high traffic and background areas.

## 2. Materials and Methods 

### 2.1. School Sampling

**Figure 1:**
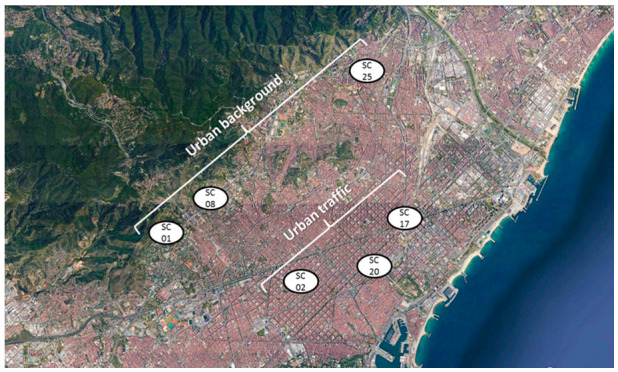
Schools selected for study in the urban background and traffic intensive areas of the Barcelona metropolitan area.

Within the BREATHE project, six schools were selected for the present study based on their geographical location [22]. Three of them were located in the traffic intensive city centre, while the other three were situated in the background area of the cities periphery (Figure 1). School names were coded as SC# for privacy. PM_2.5_ aerosols were collected daily with high volume samplers (MCV CAV and DIGITEL) on Pallflex quartz filters (QAT 150UP). Sampling was not performed simultaneously in all sites because of a limited number of high volume samplers. Aerosol collection was therefore developed weekly in school pairs, one located downtown and the other at the periphery. In each school, air was filtered simultaneously in the classroom and in the outdoor playground during four consecutive working days from 9:00 until 17:00. The whole sampling scheme was repeated twice a year to obtain data from the cold and warm seasons in all schools.

### 2.2. Organic Tracer Analysis

A quarter of each filter sample (*n* = 96) was analyzed for molecular organic markers and PAHs following the methodology described elsewhere [6,24]. The filter section for chemical analysis was Soxhlet extracted with (2:1, v/v) dichloromethane:methanol (60 mL; Merck, Germany) for 8 h. Before extraction, 25 μL of the surrogate standards d_7_-levoglucosan, d_50_-n-C_24_ (Cambridge Isotopic Laboratories, UK), d_4_-succinic acid (Sigma Aldrich), d_10_-anthracene, d_12_-benz[a]anthrancene, d_12_-benzo[k]fluoranthene and d_12_-benzo[ghi]perylene (Dr. Ehrenstorfer) were added. The extracts were filtered through glass fiber filters using a glass syringe to remove insoluble particles. Then, they were concentrated to 1 mL under a gentle nitrogen stream. 

An aliquot of the extract (25 µL) was evaporated under a gentle nitrogen stream until dry. Then, 25 μL of bis(trimethylsilyl)trifluoroacetamide (BSFTA) + trimethylchlorosilane (99:1) (Supelco) and 10 μL of pyridine (Merck) were added and heated at 70 °C during 1 h for derivatization of the saccharides, acids and polyols to trimethylsilyl esters. Before instrumental analysis, 25 µL of the internal standard, 1-phenyldodecane was added to the vial.

PAHs and hopanes were analyzed in the remaining extract that was evaporated to near-dryness under a gentle nitrogen gas stream and re-dissolved in 0.5 mL (9:1 v/v) hexane:dichloromethane (Merck, Germany). This solution was cleaned-up by an adsorption column chromatography packed with 1 g of aluminum oxide (Merck, Germany) that was activated overnight at 120 °C. The analytes were eluded with 4 mL of (9:1 v/v) hexane:dichloromethane and 4 mL of (1:2 v/v) hexane:dichloromethane, respectively (Merck, Germany). The fractions were collected together and concentrated under a gentle nitrogen gas stream to 50 µL. The internal standard, 1-phenyldodecane, was added before injection into gas chromatography coupled to mass spectrometry (GC-MS).

The sample extracts were injected into a Thermo GC-MS (Thermo Trace GC Ultra—DSQ II) equipped with a 60 m fused capillary column (HP-5MS 0.25-mm × 25-μm film thickness). The oven temperature program started at 60 °C (holding time 1 min) and was then heated to 120 °C at 12 °C/min and to 310 °C at 4 °C/min where it was held for 10 min. The injector, ion source, quadrupole and transfer line temperatures were 280 °C, 200 °C, 150 °C and 270 °C, respectively. Helium was used as a carrier gas at 0.9 mL/min^−1^. The MS detector was operated in full scan (m/z 50–650) and electron impact (70 eV) ionization mode.

Besides retention time comparison, levoglucosan, α- and β-glucose were identified with ion m/z 204, mannitol with m/z 319, sucrose and myose with m/z 361, and nicotine with ion m/z 84. Acids and polyols were identified with the following ions: succinic acid (m/z 247), glutaric acid (m/z 261), adipic acid (m/z 275) azelaic acid (m/z 317), malic acid (m/z 233), phthalic acid and terephthalic acid (m/z 295), phthalate esters (m/z 149), dihydrojasmonate (m/z 153) and galaxolide (m/z 243). Straight chain carboxylic acids were identified in the m/z 117 mass fragmentogram and the corresponding retention times. Quantification was performed with external standard calibration curves. The concentrations were corrected by the recoveries of the surrogate standard d_4_-succinic acid (m/z 251) and d_7_-levoglucosan (m/z 206). 

PAHs were identified by retention time comparison of the peaks generated with the following ions: benz[a]anthracene and chrysene+triphenylene (m/z 228), benzo[b]fluoranthene, benzo[k]fluoranthene, benzo[e]pyrene and benzo[a]pyrene (m/z 252), indeno[1,2,3-cd]pyrene and benzo[ghi]perylene (m/z 276). 17α(H)-21β(H)-29-Norhopane and 17α(H)-21β(H)-hopane were identified in the m/z 191 mass fragmentogram and the corresponding retention times. Quantification was also performed by the external standard method and the calculated concentrations were corrected by the recoveries of the above-mentioned surrogate standards: d_12_-benz[a]anthrancene (m/z 240), d_12_-benzo[k]fluoranthene (m/z 264), d_12_-benzo[ghi]perylene (m/z 288) and d_50_-n-C_24_ (m/z 66).

### 2.3. Complementary Data

The obtained data of PAH concentrations of each sample were normalized to the overall urban air pollution in Barcelona in order to be able to compare the concentrations obtained in different schools in different samplings [22]. For this purpose, the benzo[a]pyrene (BaP) concentrations measured in three urban monitoring stations of *Eixample* (41.385315 N, 2.1537998 E), *Gràcia-Sant Gervasi* (41.398724 N, 2.1533988 E) and *Plaça de la Universitat* (41.387379 N, 2.164895 E) by the air service of the Autonomous Government of Catalonia were used as reference data. These three stations are urban road sites (RS) highly exposed to traffic. BaP is measured daily at least in one of these stations. The normalized indoor and outdoor concentration ${\left(C_{i}^{j}\right)}_{k}^{*}$ of the *i* pollutant for day *k* at school *j* was calculated as described in Equation (1):

(1) $${\left(C_{i}^{j}\right)}_{k}^{*}={\left(C_{i}^{j}\right)}_{k}/\left[{\left(C^{RS}\right)}_{k}/\bar{\left(C^{RS}\right)}\right]$$

where ${\left(C_{i}^{j}\right)}_{k}$ is the concentration measured at the school, ${\left(C^{RS}\right)}_{k}$ is the average BaP concentration in the three monitoring stations on day *k* and $\bar{\left(C^{RS}\right)}$ is the average BaP concentration in the three RS stations for the whole study period.

These normalized data were only used for spatial variation analyses of the air compounds measured in each school. Comparisons and correlations between individual compound concentrations were performed with non-normalized daily data.

As described elsewhere [22,23,25], OC, determined as volatilized carbon in the absence of oxygen, and EC, measured after OC removal by pyrolysis of the filter in the presence of oxygen (10%), were determined by a thermal-optical transmission technique [26] using a Sunset Laboratory OCEC Analyzer with the NIOSH temperature program. PM_2.5_ was measured by filter weighing after blank subtraction. The overall chemical characterisation for source apportionment of the organic fraction was completed by multivariate curve resolution—alternating least-squares (MCR-ALS) analysis [27].

## 3. Results

The schools with highest pollution levels were geographically located downtown, characterized by dense and congested traffic, while the lowest concentrations were observed in the periphery of the metropolitan area (Figure 1).

### 3.1. Bulk Measurements

**Figure 2:**
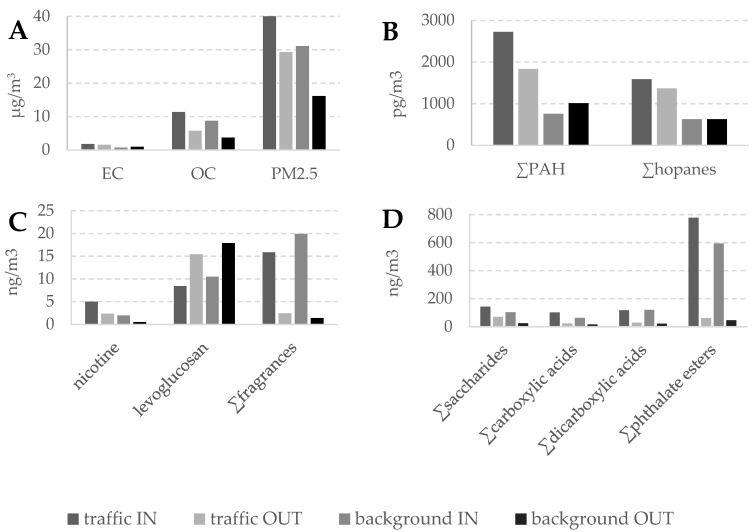
Mean concentrations of (**A**) elemental carbon (EC), organic carbon (OC), PM_2.5_, and the sum of organic molecular compounds (**B**): polycyclic aromatic hydrocarbon (PAH), hopanes, (**C**): nicotine, levoglucosan, fragrances, (**D**): saccharides, (di-)carboxylic acids, and phthalate esters) in the indoor and outdoor aerosols of the schools located near traffic and background sites.

The average concentrations of EC, OC and PM_2.5_ in the indoor and outdoor atmospheres of the schools with high and low traffic are reported in Table 1 and Figure 2. The EC concentrations were generally similar to those observed in streets in other urban areas [25,28,29]. However, a large difference was observed between the schools from the high traffic and background areas, as the indoor concentrations of the former were more than double those of the latter, 1.8 µg/m^3^ and 0.7 µg/m^3^, respectively. This traffic–background ratio was also nearly doubled in the average outdoor concentrations, 1.5 µg/m^3^ and 0.9 µg/m^3^, respectively. In contrast, the differences between indoor and outdoor average concentrations were small and did not show a specific trend: 1.8 µg/m^3^ and 1.5 µg/m^3^, respectively, in the schools from traffic areas, and 0.7 µg/m^3^ and 0.9 µg/m^3^, respectively, in the schools from background areas. Thus, the average indoor/outdoor EC ratio of all samples is 1.0 with a standard deviation of 0.4.

The average concentrations of OC (Table 1) were also higher in the schools located in the areas with intensive traffic than those situated in the urban background, 11 µg/m^3^ and 8.7 µg/m^3^, respectively, for the average indoor atmospheres, and 5.7 µg/m^3^ and 3.7 µg/m^3^ for the average indoor air, respectively, but in this case the differences were not as large as for EC. The largest OC differences were observed when comparing indoor and outdoor samples, involving nearly double or more indoor than outdoor concentration: 11 µg/m^3^ and 5.7 µg/m^3^, respectively, in the schools located in high traffic areas, and 8.7 µg/m^3^ and 3.7 µg/m^3^, respectively, in the schools from background areas (the average indoor/outdoor OC ratio of all samples is 2.2). These differences were also described in Rivas et al. [22] and were consistent with the use of organic materials within the schools, e.g., clothes, food and other items, that may release particles to ambient air. Accordingly, the OC/EC ratios of these average concentrations showed higher values indoor (6.1 and 12 in the high and low traffic schools, respectively) than outdoor (3.8 and 4.1 in the high and low traffic schools, respectively) which again is consistent with the emission of organic particles in the school buildings as consequence of the tutorial activities. Furthermore, the typical traffic pollution OC/EC ratios in urban atmospheres are about 2 [30,31] and the ratios of diesel exhaust particulates range between 1.9 and 2.3 [26]. The higher OC/EC ratios observed in the outdoor atmosphere of the schools support that organic components associated to the school activities also have an influence in the outdoor air of the school playgrounds.

Concerning PM_2.5_, higher concentrations (Table 1; Figure 2) were observed in the schools from high traffic areas, with averages of 40 µg/m^3^ and 29 µg/m^3^ indoor and outdoor, respectively, than in those from background sites, 31 µg/m^3^ and 16 µg/m^3^ indoor and outdoor, respectively, which is consistent with other studies in urban areas [32,33]. The findings in this study were described and discussed elsewhere [22,23]. In addition, the higher PM_2.5_ concentrations indoor than outdoor in both schools indicates that the above-mentioned tutorial activities involve higher airborne particles also in the inhalable size range as already indicated previously [22,23].

### 3.2. Polycyclic Aromatic Hydrocarbons

The traffic normalized concentrations of the PAHs were very similar to those measured daily ([normalized compound concentration] = [compound concentration]/1.05 + 3.84; r = 0.90, *p* < 0.01), showing that the measurements at the schools were representative of the general exposure conditions. The mean normalized indoor and outdoor concentrations of the organic compounds analyzed in the PM_2.5_ filter samples are also shown in Table 1 and Figure 2. The results are grouped by averages of the schools located in high-traffic areas and in background sites, as performed with the bulk properties. The mean concentrations of the sum of the seven PAHs in the schools ranged from 500 pg/m^3^ to 3900 pg/m^3^. These concentrations are comparable to those in other European urban areas [34]. The average outdoor concentrations in the schools from the high traffic areas and the periphery were 1800 pg/m^3^ and 1000 pg/m^3^, respectively, and the average indoor concentrations of these two school groups were 2700 pg/m^3^ and 760 pg/m^3^, respectively. The similarity of the average indoor and outdoor concentrations of the heavier molecular weight PAH, e.g., benzofluoranthenes, benzopyrenes, indeno[1,2,3-cd]pyrene and benzo(ghi)perylene (1200–1300 pg/m^3^ in high traffic areas and 520–670 pg/m^3^ in the periphery), was consistent with the transfer of outdoor traffic emissions to the indoor classroom ambient air.

Benzo[a]pyrene (BaP), the only PAH with a regulated target concentration of 1000 pg/m^3^ [35], had a total mean indoor and outdoor concentration of approximately 70 pg/m^3^, and ranged between 20 pg/m^3^ and 170 pg/m^3^ in the different schools. These BaP concentrations are in the range of those measured in outdoor PM in the monitoring stations for air quality in the city of Barcelona (mean BaP = 130 pg/m^3^, ranging from 70 pg/m^3^ in an urban background site and 200 pg/m^3^ in traffic sites). Comparison of these data with those collected 25 years ago in an urban traffic site of Barcelona shows that the past concentrations were much higher, 80,000 pg/m^3^ for the total PAH and 11,000 pg/m^3^ for BaP [36]. These former concentrations are nowadays observed in Asian urban areas, such as those in Xi’an, China (26,000 pg/m^3^; [37]) or Dehli, India (78,000 pg/m^3^; [38]), which are related to inefficient biomass and coal combustion and exhausts from high traffic density.

The benzo[b+j+k]fluoranthenes (27% of ∑PAH) were the predominant PAHs in the outdoor air mixtures, followed by chrysene (24%) and benzo[ghi]perylene (18%). The indoor air had higher contributions of the more volatile PAH, e.g., benz[a]anthracene (14% of ∑PAH) and chrysene (34%). In general, the average indoor and outdoor PAH concentrations were very similar (ratio of average indoor/outdoor ΣPAH = 1.2 ± 0.4), which, as observed for the EC, is consistent with an external source, such as inputs from nearby streets. In this respect, the average concentrations of the PAH compounds and EC content are linearly correlated (0.85 < r < 0.95; *p* < 0.01). On the other hand, the higher proportion of benz[a]anthracene and chrysene+triphenylene could reflect enhanced gas-to-particle phase partitioning due to the higher OC levels indoor, favouring the partitioning of the more volatile PAH from gas to particulate phase [39]. Hence, benz[a]anthracene and chrysene+triphenylene showed significant correlations with OC, r = 0.63 and 0.60 (*p* < 0.05), respectively, while the correlations with the other PAHs were not significant (r < 0.41; *p* > 0.05).

### 3.3. Organic Molecular Tracers

The average concentrations of the organic molecular tracers measured in the school PM_2.5_ aerosols are also shown in Table 1 and Figure 2. The hopanes are molecular markers of mineral oils, whose occurrence in atmospheric samples can be related to unburned lubricating oil residues from primary vehicle emissions [40]. Accordingly, they are found in much higher average concentrations in the schools located in the high traffic area (1600 pg/m^3^ and 1400 pg/m^3^ indoor and outdoor, respectively) than in the background area (600 pg/m^3^ both indoor and outdoor). These school concentrations are similar to those found in previous studies of street environments from Barcelona [6].

Levoglucosan, a tracer compound for biomass burning [41], shows higher concentrations in the outdoor school air and no difference between traffic and background areas (Table 1; Figure 2). Overall, the observed concentrations, 8–18 ng/m^3^, are very low in relation to previous descriptions in Europe [42] which indicates that this source is not significant in the schools and is in agreement with former studies in the Metropolitan area of Barcelona [43,44].

Nicotine, a tracer compound for cigarette smoke [45], has overall low concentrations (1–5 ng/m^3^), although the levels are two times higher indoor than outdoor and two times higher in schools situated near traffic sites (Table 1; Figure 2). This trend is comparable to former analysis in the urban area of Barcelona, where the average nicotine concentrations were 58 ng/m^3^ in an urban road site, while they were 7 ng/m^3^ in an urban background site [6]. In any case, the Table 1 results show that the contributions of cigarette smoking to the school atmospheres are negligible, even in those situated in the area of high traffic.

The occurrence of saccharides in aerosols (Table 1; Figure 2), such as α- and β-glucose (mono-saccharides), mannitol (alcohol sugar), sucrose and mycose (polysaccharides) has been related to dust or soil inputs as they are tracer compounds for vegetal debris and fungi [46,47]. However, several of these compounds, e.g., sucrose, glucoses, mannitol, may be used in indoor environments and therefore contribute to the overall burden of atmospheric particles. This was likely the case in the schools considered for study in view of the predominance of the compounds usually related to sugar-holding food consumption, e.g., sucrose and glucoses. These compounds, as well as mannitol, another sweetener, were found in much higher concentrations indoor than outdoor. Conversely, as markers of soil contributions, higher concentrations in the atmosphere of the school playgrounds was expected. In this respect, mycose, a known polysaccharide mainly associated to fungal inputs, is found in similar concentrations indoor and outdoor (Table 1).

Similar indoor–outdoor trends were observed for the carboxylic acids (Table 1; Figure 2). These acids, saturated and unsaturated, are tracer compounds of biological materials, including vegetation, oils and human tissues [48]. In the schools considered in the present study, palmitic acid (C_16:0_) was the most abundant fatty acid, followed by stearic acid (C_18:0_). Oleic acid (C_18:1_), an unsaturated acid mostly related to food cooking with oils, was much lower than C_18:0_, indicating that fresh cooking emissions were not an important source for the indoor and outdoor PM [48]. The higher concentrations of these fatty acids indoor than outdoor is consistent with contributions related to food, skin tissue or personal care products to the PM_2.5_ aerosols of the schools.

Concerning the group of dicarboxylic and hydroxy dicarboxylic acids, malic acid is a food constituent and used as food additive. Azelaic acid is present in wheat, rye and barley, and also found in personal care products. Adipic acid is a constituent of nylon. Glutaric and succinic acids have different metabolic functions. Malic and azelaic acids are the dicarboxylic/hydroxydicarboxylic acids present in higher concentrations in the air particles of both types of schools (Table 1; Figure 2) which is consistent with the food-related inputs observed with saccharides and fatty acids. The higher occurrence of these acids indoor than outdoor is also consistent with contributions of food to the school aerosols. The higher air concentrations of these acids indoor than outdoor allows excluding a photochemical origin as observed in other environments [49,50,51].

Phthalate esters are plasticizers in a large variety of polymers [52]. Among 16 phthalate esters analyzed, only diisobutyl phthalate (DIBP), di-n-butyl phthalate (DBP) and dicyclohexyl phthalate (DCHP) were identified above limit of detection. The most abundant compound was DIBP. These compounds were found in higher concentrations indoor than outdoor which probably reflects the use of plastics in classroom material (Table 1; Figure 2). These inputs were not related to traffic contributions, as both schools from high-traffic and background areas showed similar concentrations of phthalic acid, DiBP and DCHP. Schools with higher DBP in the air particles likely reflect older classroom material, since DIBP has been substituting DBP as plasticizer in many products [53].

The synthetic fragrances, methyl dihydrojasmonate and galaxolide, are used in perfumes and flavors [54]. These compounds were also found in the PM_2.5_ particles of the schools (Table 1; Figure 2). They were found in higher concentrations indoor than outdoor. The observed indoor concentrations, 10–15 ng/m^3^ and 5–6 ng/m^3^ of methyl dihydrojasmonate and galaxolide, respectively, were similar to those found in indoor air of subway stations in the city [55], and the galaxolide levels were also comparable with those reported in kindergartens and primary schools from Germany and Turkey [56,57].

### 3.4. Source Apportionment of Organic Aerosol

Further insight into the contributions of the diverse compound sources to the outdoor and indoor organic aerosols in these schools was obtained by application of the MCR-ALS method [58] to the observed sample concentrations. This method already provided useful results in source apportionment of organic aerosols from urban and rural areas [6,7]. MCR-ALS is based on an alternating linear least-squares optimization under non-negativity constraints which produces better physically-significant profiles than principal component analysis and has been shown to yield results analogous to positive matrix factorization [27].

**Figure 3:**
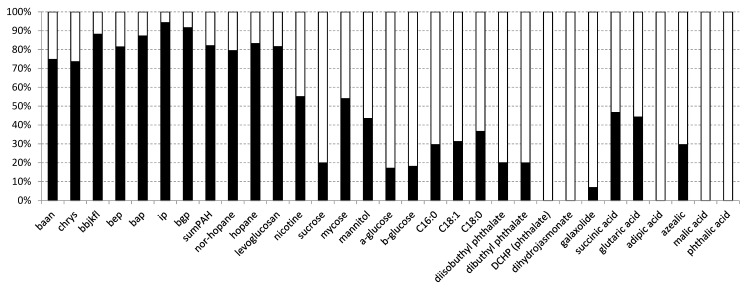
Relative component loadings (%) obtained from multivariate curve resolution-alternating least squares of the organic molecular tracer compounds in PM_2.5_ aerosols from the schools shown in Figure 1 that represent the indoor (white bars) and outdoor (black bars) organic aerosol (OA). The abbreviations in the abscissas axis are indicated in Table 1.

This MCR-ALS approach provided two components that explained 91% of the concentration variance of the organic compounds present in the indoor and outdoor PM_2.5_ of the schools (Figure 3). The loadings showed a first component that was dominated by PAHs, hopanes and levoglucosan and represented about 73–95% of the variance of the outdoor organic aerosols. The predominance of PAHs and hopanes indicated that this component was related with traffic inputs.

The second component was dominated by phthalates, sucrose, glucoses, galaxolide, methyl dihydrojasmonate, phthalic, adipic, malic and azelaic acids. The fatty acids were also significant constituents but with lower loadings (Figure 3). This component was related with food, plastics, fragrances and other elements that may be used in schools and generate airborne particles. It represented 80–100% of the variance of the indoor organic aerosols.

However, some organic compounds were not well resolved between these two components, e.g., nicotine, mycose, mannitol, succinic and glutaric acids, indicating that they originated from multiple sources that are present in both the outdoor and indoor environments (Figure 3).

The sum of the MCR-ALS scores of the indoor and outdoor organic aerosols components were correlated with the EC (r = 0.60; *p* < 0.01), OC (r = 0.91; *p* < 0.01) and PM_2.5_ (r = 0.92; *p* < 0.01), indicating that the variability of the studied organic molecular tracers nearly reflected the total aerosol variability.

**Figure 4:**
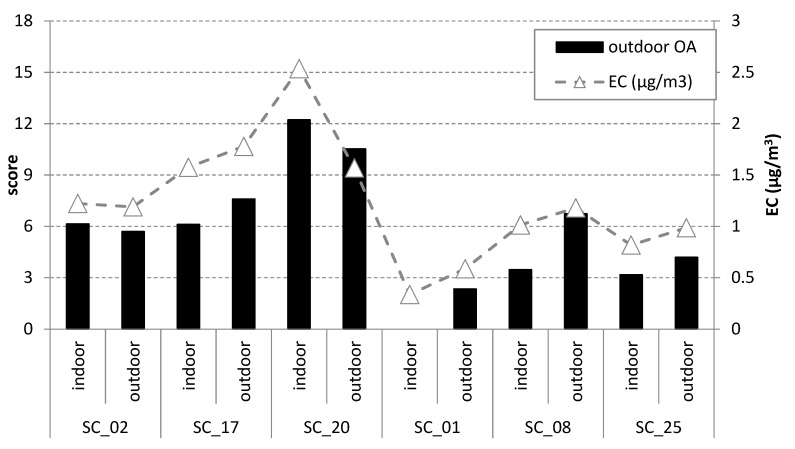
Score values of the outdoor organic aerosol component obtained by positive matrix factorization of the concentrations of the organic molecular tracers of the selected schools (left *y*-axis). The average EC concentration in the schools (right *y*-axis; µg/m^3^) is also displayed. The locations of the stations are shown in Figure 1.

The score values of the first (outdoor organic aerosols) component in the schools were strongly correlated with the EC concentrations (Figure 4; r = 0.93; *p* < 0.01). This high correlation coefficient indicated that the EC variability was associated with traffic inputs which determined the concentrations of PAHs in both playground and classroom PM_2.5_ aerosols. The correlation of the outdoor organic aerosol component is also correlated with PM_2.5_ (r = 0.36; *p* < 0.01), although weaker, while there was no correlation between the score values of this component and the OC concentrations (r = 0.15; *p* > 0.1), indicating that the outdoor organic aerosol component has a weaker influence on the total OC and PM_2.5_ concentrations.

**Figure 5:**
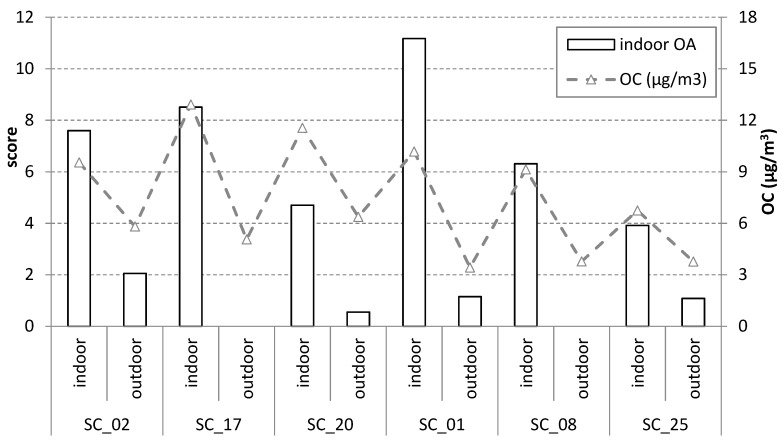
Scores values of the two components, interpreted as outdoor and indoor organic aerosol (left *y*-axis), obtained by positive matrix factorization of the concentrations of organic molecular tracers of the selected schools. The average OC concentration in the schools (right *y*-axis, µg/m^3^) is also displayed. The locations of the stations are shown in Figure 1.

On the other hand, the score values of the second (indoor organic aerosol) component in the schools indicates the peak concentrations of the OC (Figure 5), pointing out to the strong influence of the classroom activities and material in the generation of the indoor organic aerosols and therefore as sources of indoor OC content. There is a strong correlation of the score values of the indoor organic aerosol component and the OC concentrations (r = 0.84; *p* < 0.01) and the PM_2.5_ concentrations (r = 0.63; *p* < 0.01), while the correlation with EC is weak (r = −0.21; *p* < 0.05).

## 4. Conclusions

Similar PAH concentrations were found in the indoor and outdoor PM_2.5_ aerosols collected in the urban schools of Barcelona. Traffic emissions were the main contributors of these compounds in the air of both classrooms and playgrounds, with about two to three times-higher levels in the schools located in the city areas with high traffic. The similarity of the average indoor and outdoor concentrations of the higher molecular weight and more particulate-bounded PAH and EC was consistent with the transfer of outdoor traffic emissions to the indoor classroom ambient air.

The concentrations of benzo[a]pyrene did not exceed the legal annual target value, but in one of them they were higher than 120 pg/m^3^, a World Health Organization [59] guideline value above which adverse health effect could be generated.

Multivariate analysis showed that the concentrations of these aromatic compounds were correlated with those of hopanes, which are present in lubricating oils. This result confirmed the dominance of traffic as the main source for the occurrence of these hydrocarbons both in the indoor and outdoor atmospheres of the schools. EC was also correlated with these compounds, showing that traffic pollution was the main determinant of this aerosol carbonaceous fraction.

The concentrations of glucoses, sucrose, malic, azealic and fatty acids were correlated and grouped in the same multivariate component, showing that these food-related markers were present in higher levels in the indoor atmospheres. Other markers of domestic activity such as phthalate esters, additives of plastic materials, adipic acid, a nylon constituent, and fragrances, galaxolide and methyl dihydrojasmonate, from cleaning and personal care products, were also correlated with the food-stuff tracers. All of these compounds were found in higher amounts in the indoor atmospheres and correlated with the measured OC fraction of the aerosols.

In order to reduce the ambient concentrations of PAH, the intensity of traffic around the schools should be reduced, especially in the city centre. For the reduction of indoor particles, dust could be collected more often, while cleaning products could be checked for the presence of undesirable ingredients. Moreover, the classroom material could be checked for the composition of undesirable plasticizers, such as DCHP and DBP.

## Figures and Tables

**Table 1:** Mean concentrations and standard deviations of organic tracer compounds in the indoor and outdoor aerosols of the schools located near traffic and background sites.

		Traffic Indoor (*n* = 24)	Traffic Outdoor (*n* = 24)	Background Indoor (*n* = 24)	Background Outdoor (*n* = 24)
µg/m^3^	Elemental Carbon	1.8 ± 0.7	1.5 ± 0.3	0.7 ± 0.3	0.9 ± 0.3
	Organic Carbon	11 ± 1.7	5.7 ± 0.7	8.7 ± 1.8	3.7 ± 0.2
	PM_2.5_	40 ± 8	29 ± 4.9	31 ± 7.8	16 ± 3.2
pg/m^3^	Benz[a]anthracene (baan)	440 ± 140	190 ± 97	60 ± 22	81 ± 53
	Chrysene + triphenylene (chrys)	990 ± 300	440 ± 160	180 ± 61	260 ± 120
	Benzo[bjk]fluoranthenes (bbjkfl)	540 ± 240	480 ± 130	210 ± 90	280 ± 170
	Benzo[e]pyrene (bep)	180 ± 50	140 ± 47	63 ± 20	76 ± 41
	Benzo[a]pyrene (bap)	110 ± 55	86 ± 43	34 ± 11	36 ± 21
	Indeno[1,2,3-cd]pyrene (ip)	145 ± 73	160 ± 78	74 ± 24	100 ± 61
	Benzo[ghi]perylene (bgp)	330 ± 150	330 ± 120	140 ± 54	180 ± 110
	17α(H),21β(H)-29-nor-Hopane (nor hopane	960 ± 340	820 ± 83	390 ± 160	380 ± 220
	17α(H),21β(H)-Hopane (hopane)	630 ± 260	545 ± 37	230 ± 91	250 ± 160
ng/m^3^	Levoglucosan	8 ± 5	15 ± 5	10 ± 3	18 ± 13
	Nicotine	5 ± 2	2 ± 2	2 ± 1	1 ± 1
	Sucrose	66 ± 51	39 ± 57	34 ± 14	7 ± 5
	Mycose	3 ± 1	4 ± 3	2 ± 1	1 ± 0
	Mannitol	12 ± 2	6 ± 1	6 ± 3	4 ± 2
	α-Glucose	29 ± 11	10 ± 6	29 ± 21	6 ± 4
	β-Glucose	32 ± 12	11 ± 7	32 ± 22	8 ± 5
	Hexadecanoic acid (C16:0)	66 ± 10	15 ± 6	41 ± 18	10 ± 3
	Octadecenoic acid (C18:1)	8 ± 2	2 ± 0	5 ± 2	1 ± 0
	Octadecanoic acid (C18:0)	27 ± 9	7 ± 3	16 ± 7	5 ± 1
	Succinic acid	7 ± 2	5 ± 2	6 ± 2	4 ± 1
	Glutaric acid	2 ± 0	1 ± 1	2 ± 1	1 ± 0
	Adipic acid	6 ± 1	2 ± 1	10 ± 11	1 ± 0
	Azealic acid	26 ± 4	9 ± 1	22 ± 6	6 ± 2
	Malic acid	25 ± 2	7 ± 2	38 ± 43	6 ± 1
	Phthalic acid	51 ± 33	3 ± 1	42 ± 42	3 ± 1
	Diisobutyl Phthalate	410 ± 51	38 ± 20	390 ± 110	28 ± 18
	Dibutyl Phthalate	270 ± 120	12 ± 8	92 ± 29	9 ± 7
	Dicyclohexyl Phthalate (DCHP)	95 ± 13	12 ± 3	110 ± 93	9 ± 3
	Methyl Dihydrojasmonate	10 ± 4	1 ± 1	15 ± 6	1 ± 1
	Galaxolide	6 ± 2	1 ± 1	5 ± 2	1 ± 0
